# Insights into time fractional dynamics in the Belousov-Zhabotinsky system through singular and non-singular kernels

**DOI:** 10.1038/s41598-023-49577-1

**Published:** 2023-12-15

**Authors:** Shami A. M. Alsallami, M. Maneea, E. M. Khalil, S. Abdel-Khalek, Khalid K. Ali

**Affiliations:** 1 Mathematics Department, College of Sciences, Umm Al-Qura University, Makkah, 24381 Saudi Arabia; 2 Faculty of Engineering, MTI University, Cairo, Egypt; 3 Department of Mathematics, College of Science, Taif University, P.O.Box 11099, Taif, 21944 Saudi Arabia; 4 Mathematics Department, Faculty of Science, Al-Azhar University, Nasr-City, Cairo, Egypt

**Keywords:** Mathematics and computing, Optics and photonics

## Abstract

In the realm of nonlinear dynamics, the Belousov-Zhabotinsky reaction system has long held the fascination of researchers. The Belousov-Zhabotinsky system continues to be an active area of research, offering insights into the fundamental principles of nonlinear dynamics in complex systems. To deepen our understanding of this intricate system, we introduce a pioneering approach to tackle the time fractional Belousov-Zhabotinsky system, employing the Caputo and Atangana-Baleanu Caputo fractional derivatives with the double Laplace method. The solution we obtained is in the form of series which helps in investigating the accuracy of the proposed method. The primary advantage of the proposed technique lies in the low amount of calculations required and produce high degree of precision in the solutions. Furthermore, the existence and uniqueness of the solution are investigated thereby enhancing the overall credibility of our study. To visually represent our results, we present a series of 2D and 3D graphical representations that vividly illustrate the behavior of the model and the impact of changing the fractional order derivative and the time on the obtained solutions.

## Introduction

Fractional calculus, a branch of mathematical analysis that generalizes the concept of differentiation and integration to non-integer orders, has gained increasing prominence in various scientific disciplines over the past few decades. Initially introduced by Leibniz in the 17th century and developed by several mathematicians in subsequent centuries^1,2^. Fractional calculus is now recognized as a powerful tool for modeling and analyzing complex phenomena with memory and non-local behaviors, it finds extensive applications in a wide range of engineering and scientific domains, encompassing fields such as chemistry, physics, economics, biology, and finance^3–7^. There are many definitions of fractional calculus, including, the Riemann-Liouville ($\textsf{RL}$) definition focuses on historical memory and is suitable for analyzing processes with memory effects^8^. On the other hand, the Caputo definition incorporates initial conditions, making it particularly useful for modeling systems with specific starting conditions^9,10^, Riesz fractional derivative is often used in physics and engineering, especially in the context of fractional diffusion and wave equations^11,12^. Caputo-Fabrizio ($\textsf{CF}$)derivative has been arising recently to overcome the singularity on the integral kernel^13,14^. The Atangana-Baleanu ($\textsf{AB}$) fractional derivative is a relatively recent development in fractional calculus, it is known for its capability to effectively capture memory effects and non-local behavior, making it suitable for various applications^15^, and many other definitions, each of which has its distinguishing characteristics. Solving nonlinear problems with fractional orders can pose greater challenges due to the involvement of integral operators. Nevertheless, various computational approaches have been devised and applied to explore both precise and numerical solutions for such fractional problems^16–18^. Fractional systems of partial differential equations(PDEs) have garnered significant attention because of their ability to effectively represent intricate phenomena that go beyond the descriptive capacity of traditional integer-order models^19,20^, this is because, unlike ordinary derivatives, which describe the rate of change of a function at a specific point, fractional derivatives account for the past behavior of the function over a range of values. They are known to capture non-local and memory effects, which means they can take into account the entire history of a function rather than just its current value. This property enables fractional derivatives to describe the real life applications.

In this study, we aim to investigate the time fractioal Belousov-Zhabotinsky system (TFBZS) in the form:

1 $$\begin{aligned} \begin{aligned}{}&\frac{\partial ^\theta \textsf{p} }{\partial t^\theta }= \zeta _1 \frac{\partial ^{2} \textsf{p} }{\partial x^{2}}+\beta \delta \textsf{w}+\textsf{p}-\textsf{p}^2 -\delta \textsf{p} \textsf{w},\\&\frac{\partial ^\theta \textsf{w} }{\partial t^\theta }=\zeta _2 \frac{\partial ^{2} \textsf{w} }{\partial x^{2}}+\gamma \textsf{w} -\lambda \textsf{p} \textsf{w}. \end{aligned} \end{aligned}$$

Constrained by the starting conditions:

$$\begin{aligned} \begin{aligned}{}&\textsf{p}(x,0)=\textsf{p}_0(x,t),\\&\textsf{w}(x,0)=\textsf{w}_0(x,t). \end{aligned} \end{aligned}$$

Where $\theta$ is the fractional order derivative $0 <\theta \le 1$. $\textsf{p}(x,t)$ and $\textsf{w}(x,t)$ are the concentration in chemical reaction as a function of temporal oscillations and spatial traveling concentration waves. $\zeta _1$ and $\zeta _2$ represent the diffusing constants, in this study, we consider $\zeta _1=\zeta _2=1$, $\beta , \delta , \gamma$ and $\lambda$ are positive constants and $\lambda \ne 1$. Due to the importance of this system, many researchers have dealt with this system, whether using fractional calculus or ordinary calculus, to obtain exact and approximate solutions, Ali Jaradat et al. present a numerical solution for the TFBZS using generalized Taylor series^21^. Akinyemi^22^ investigted the TFBZS using q-homotopy analysis transform method. Alaoui et al.^23^ provide an approximate solution for the proposed system using homotopy perturbation method with Yang transform. Karaagac et al.^24^applied the Picard-Lindelöf theorem to the proposed model under $\textsf{ABC}$ fractional derivative. Veeresha^25^ presented a brief analysis for this chemical reaction under Caputo fractional derivative. Recently, El-Tantawy et al.^26^ presented an approximate solution for the TFBZS using residual power series merged with Laplace transform. Yasmin et al.^27^ investigated this system using homotopy perturbation with Elzaki transform.

In this study, we use the double Laplace method (DLM) merged with Adomian polynomials to enable us deal with nonlinear terms^28–30^ under two types of fractional derivatives, Caputo ($\textsf{C}$) and Atangana-Baleanu-Caputo ($\textsf{ABC}$) fractional derivatives.

The structure of this article is as follows: In section “Basic definitions”, we provide an overview of the essential concepts employed to solve the proposed equation, including fractional derivatives and integrals, as well as Adomian polynomials. In section “Framework of Double Laplace method”, we introduce the framework of the DLM in the sense of $\textsf{C}$ and $\textsf{ABC}$ fractional derivative as a method for finding an approximate solution. In section “Analysis of the existence and uniqueness of the solution”, we delve into discussions regarding the existence, and uniqueness of the solution. Section “Solution of the TFBZS using DLM” offers a concise explanation of the solution of the TFBZS using DLM. The outcomes derived in section “Solution of the TFBZS using DLM” are visually represented in section “Graphic representations”. Finally, the concluding remarks of this study are presented in section “Conclusion”.

## Basic definitions

### Fractional derivatives

As we mentioned earlier, there are many definitions of fractional differentiation, each of them has its own advantages and disadvantages. It’s important to highlight that Caputo’s definition is applicable solely to functions that are differentiable. In 2016 Abdon Atangana and Dumitru Baleanu presented a new fractional derivative with non-local and no-singular kernel that depend on Mittag-Leffler function^31^. The studies conducted in recent years following these advancements clearly indicate that scientists have a significant opportunity to address a variety of issues using fractional derivatives.

In this research, our objective is to solve TFBZS using DLM in the sense of Caputo and ABC fractional derivative.

#### Definition 2.1

^2^: Caputo fractional derivative is defined as:

2 $$\begin{aligned} {}^ \textsf{C} \mathscr {D}_{ t }^{\theta } \mathcal {F}( t )= {\left\{ \begin{array}{ll} \jmath ^{ p -\theta }\dfrac{\textsf{d}^{ p }}{\textsf{d} t ^{ p }}\mathcal {F}( t ), \, &{} p -1<\theta < p ,\\ \\ \dfrac{\textsf{d}^{ p }}{\textsf{d} t ^{ p }} \mathcal {F}( t ), &{} \theta = p . \end{array}\right. } \end{aligned}$$

$\jmath ^{ p -\theta }$ represents the $\textsf{RL}$ fractional integral in the form:

3 $$\begin{aligned} \jmath ^{\theta } \mathcal {F}( t )=\dfrac{1}{\Gamma (\theta )}\,\,\ \int ^{ t }_{0}( t -\textsf{T})^{(\theta -1)}\,\, \mathcal {F}(\textsf{T}) \,\, \textsf{d}\textsf{T}, \,\,\,\,\, t >0. \end{aligned}$$

$\Gamma (.)$ is the known Gamma function.

The operator $\jmath ^{\theta }$ satisfy the following properties for $\mathcal {E},\mathcal {G} \ge -1$:

4 $$\begin{aligned} \jmath ^{\mathcal {G}} \jmath ^{\mathcal {E}}\mathcal {F}( t )= & {} \jmath ^{\mathcal {G}+\mathcal {E}}\mathcal {F}( t ). \end{aligned}$$

5 $$\begin{aligned} \jmath ^{\mathcal {G}} \jmath ^{\mathcal {E}}\mathcal {F}( t )= & {} \jmath ^{\mathcal {E}} \jmath ^{\mathcal {G}}\mathcal {F}( t ). \end{aligned}$$

6 $$\begin{aligned} \jmath ^{\mathcal {G}} t ^{\mathfrak {m}}= & {} \frac{\Gamma (\mathfrak {m}+1)}{\Gamma (\mathfrak {m}+1+\mathcal {G})} t ^{\mathfrak {m}+\mathcal {G}}. \end{aligned}$$

Caputo fractional derivative satisfy:

7 $$\begin{aligned}{} & {} {}^ \textsf{C} \mathscr {D}_{ t }^{\theta } \bigg [\jmath ^{\theta }\mathcal {F}( t ) \bigg ]=\mathcal {F}( t ). \end{aligned}$$

8 $$\begin{aligned}{} & {} \jmath ^{\theta } \bigg [\, ^\textsf{C} \mathscr {D}_{ t }^{\theta } \mathcal {F}( t )\bigg ]= \mathcal {F}( t )- \sum _{\mathfrak {n}=0}^{\textsf{p}-1}{\mathcal {F}^{\mathfrak {n}}(0)} \frac{ t ^{\mathfrak {n}}}{\mathfrak {n}!},\,\,\, t >0. \end{aligned}$$

9 $$\begin{aligned}{} & {} {}^ \textsf{C} \mathscr {D}_{ t }^{\theta } t ^{\textsf{m}}=\frac{\Gamma (\textsf{m}+1)}{\Gamma (\textsf{m}+1-\theta )} t ^{\textsf{m}-\theta }. \end{aligned}$$

#### Definition 2.2

^32^: $\textsf{ABC}$ fractional integral is in the form:

10 $$\begin{aligned} ^\textsf{ABC}_\textsf{a} \jmath _{ t }^{\theta } \mathcal {F}( t )=\dfrac{1-\theta }{{\mathcal {N}(\theta )}}\mathcal {F}( t ) + \dfrac{\theta }{{\mathcal {N}(\theta )}\Gamma (\theta )} \int ^{\textsf{t}}_\textsf{a} \mathcal {F}(\textsf{T})( t -\textsf{T})^{\theta -1}\,\,{\textsf{d}}\textsf{T}. \end{aligned}$$

${\mathcal {N}(\theta )}$ represents the normalization function in which ${\mathcal {N}(0)}= {\mathcal {N}(1)}=1$.

In this study, for simplicity, we will consider ${\mathcal {N}(\theta )}=1$.

#### Definition 2.3

^32^: $\textsf{ABC}$ fractional derivative is in the form:

11 $$\begin{aligned} ^\textsf{ABC} \mathscr {D}^{\theta } \mathcal {F}( t )=\dfrac{{\mathcal {N}(\theta )}}{1-\theta }\,\,\ \int ^{\textsf{t}}_\textsf{a} \mathcal {F}^\prime (\textsf{T})\, E_\theta \bigg (\frac{-\theta ( t -\textsf{T})^\theta }{1-\theta } \bigg )\,\, \textsf{d}\textsf{T}. \end{aligned}$$

Further details in different types of fractional derivatives in^33–36^.

### Adomian polynomials

The Adomian decomposition technique has introduced the concept that the unknown linear function $\mathcal {Q}$ can be represented through a sequence of decompositions:

12 $$\begin{aligned} \mathcal {Q}=\sum _{\mathfrak {j}=0}^{\infty } \mathcal {Q}_\mathfrak {j}. \end{aligned}$$

The elements $\mathcal {Q}_\mathfrak {j}$ can be recursively calculated, and the nonlinear term $\mathcal {F}(\mathcal {Q})$, which could include expressions like $\mathcal {Q}^2, \mathcal {Q}^3, \sin \mathcal {Q}, exp(\mathcal {Q})$, etc. can be represented using Adomian polynomials $(\mathcal{A}\mathcal{P})$ denoted as $\mathcal {A}_\mathfrak {j}$ within the structure:

13 $$\begin{aligned} \mathcal {F}(\mathcal {Q})=\sum _{\mathfrak {j}=0}^{\infty } \mathcal {A}_\mathfrak {j}(\mathcal {Q}_0, \mathcal {Q}_1,\ldots ,\mathcal {Q}_\mathfrak {j}). \end{aligned}$$

The calculation of $\mathcal{A}\mathcal{P}$ is used to handle different forms of nonlinearity. Adomian^37^ introduced a technique for computing $\mathcal{A}\mathcal{P}$, which has been formally validated. Other methods based on Taylor series have also been developed, as discussed in^38,39^. To compute the $\mathcal{A}\mathcal{P}$, $\mathcal {A}_\mathfrak {j}$ for the nonlinear term $\mathcal {F}(\mathcal {Q})$, you can apply the following general formula:

14 $$\begin{aligned} \mathcal {A}_\mathfrak {j}= \frac{1}{\mathfrak {j} \, !}\frac{\textsf{d}^\mathfrak {j}}{\textsf{d}\nu ^\mathfrak {j}} \bigg [\mathcal {F} \bigg ( \sum _{\ell =0}^{\mathfrak {j}} \nu ^\ell \mathcal {Q}_\ell \bigg )\bigg ]_{\nu =0}, \,\,\,\,\,\,\,\,\,\,\,\ \mathfrak {j}=0,1,2,\ldots . \end{aligned}$$

Expression (14) can be expanded as follows:

15 $$\begin{aligned} \begin{aligned}{}&\mathcal {A}_0= \mathcal {F}(\mathcal {Q}_0), \\&\mathcal {A}_1= \mathcal {Q}_1 \mathcal {F}^\prime (\mathcal {Q}_0),\\&\mathcal {A}_2= \mathcal {Q}_2\mathcal {F}^\prime (\mathcal {Q}_0)+\frac{1}{2!} \mathcal {Q}_1 ^2 \mathcal {F}^{\prime \prime }(\mathcal {Q}_0) , \\&\mathcal {A}_3= \mathcal {Q}_3 \mathcal {F}^\prime (\mathcal {Q}_0)+\mathcal {Q}_1 \, \mathcal {Q}_2 \mathcal {F}^{\prime \prime }(\mathcal {Q}_0)+\frac{1}{3!} \mathcal {Q}_1 ^3 \mathcal {F}^{\prime \prime \prime }(\mathcal {Q}_0) ,\\&:\end{aligned} \end{aligned}$$

From the relations presented in (15), we notice that $\mathcal {A}_0$ depends only on $\mathcal {Q}_0$, $\mathcal {A}_1$ depends only $\mathcal {Q}_0$ and $\mathcal {Q}_1$, $\mathcal {A}_2$ depends only on $\mathcal {Q}_0$,$\mathcal {Q}_1$ and $\mathcal {Q}_2$, etc.

## Framework of Double Laplace method

The double Laplace transform method serves as a valuable mathematical tool for addressing fractional nonlinear equations or systems of equations. This technique proves particularly effective when dealing with equations featuring Caputo, Caputo-Fabrizio, or Atangana-Baleanu-Caputo fractional derivatives. By applying the Laplace transform twice, it enables the conversion of intricate fractional differential equations into more accessible algebraic forms. This transformation simplifies the process of solving fractional differential equations. Moreover, it can be combined with methods like Adomian polynomials, see^32,40^ to effectively handle nonlinear terms within these equations. This approach significantly enhances our capability to analyze and solve real-world problems across various scientific and engineering domains.

### Definition 3.1

^32^ The expression for the Double Laplace transform using Caputo fractional derivative when $p-1 <\theta \le p$ can be described as follows:

16 $$\begin{aligned}{} & {} \mathscr {L}_x \mathscr {L}_t \bigg \{\ \,^\textsf{C}\mathscr {D}_{x }^{\theta } \mathcal {F}(x,t )\bigg \} = S _1^\theta \,\, \mathcal {F}( S _1, S _2)-\sum _{\mathfrak {j}=0}^{ p-1 } S _1^{\theta -1-\mathfrak {j}}\mathscr {L}_t \bigg \{\frac{\partial ^\mathfrak {j}\mathcal {F}(0,t ) }{\partial x^\mathfrak {j} }\bigg \}, \end{aligned}$$

17 $$\begin{aligned}{} & {} \mathscr {L}_x \mathscr {L}_t \bigg \{ \,^\textsf{C}\mathscr {D}_{t }^{\theta } \mathcal {F}(x,t )\bigg \} = S _2^\theta \,\, \mathcal {F}( S _1, S _2)-\sum _{\mathfrak {j}=0}^{ p-1 } S _2^{\theta -1-\mathfrak {j}}\mathscr {L}_x \bigg \{\frac{\partial ^\mathfrak {j}\mathcal {F}(x,0) }{\partial t^\mathfrak {j} }\bigg \}. \end{aligned}$$

### Definition 3.2

^32^ The expression for the Double Laplace transform using Atangana-Baleanu-Caputo fractional derivatives when $p-1 <\theta \le p$ can be described as follows:

18 $$\begin{aligned}{} & {} \mathscr {L}_x \mathscr {L}_t \bigg \{\ \,^\textsf{ABC}\mathscr {D}_{x }^{\theta } \mathcal {F}(x,t )\bigg \} = \frac{\mathcal {N}(\theta )}{(1-\theta ) \left( S _1^\theta +\frac{\theta }{1-\theta }\right) }\bigg \{ S _1^\theta \,\, \mathcal {F}( S _1, S _2)-\sum _{\mathfrak {j}=0}^{ p-1 } S _1^{\theta -1-\mathfrak {j}}\,\mathscr {L}_t \frac{\partial ^\mathfrak {j}\mathcal {F}(0,t ) }{\partial x^\mathfrak {j} }\bigg \}, \end{aligned}$$

19 $$\begin{aligned}{} & {} \mathscr {L}_x \mathscr {L}_t \bigg \{ \,^\textsf{ABC}\mathscr {D}_{t }^{\theta } \mathcal {F}(x,t )\bigg \} =\frac{\mathcal {N}(\theta )}{(1-\theta ) \left( S _2^\theta +\frac{\theta }{1-\theta } \right) }\bigg \{ S _2^\theta \,\, \mathcal {F}( S _1, S _2)-\sum _{\mathfrak {j}=0}^{ p-1 } S _2^{\theta -1-\mathfrak {j}}\,\mathscr {L}_x \frac{\partial ^\mathfrak {j}\mathcal {F}(x,0 ) }{\partial t^\mathfrak {j} }\bigg \}, \end{aligned}$$

for $$p =1,2,3,\ldots$$.

## Analysis of the existence and uniqueness of the solution

In this section, we will establish the existence and the uniqueness of the TFBZS within the context of the $\textsf{ABC}$ sense. To do so, let’s rewrite the couple sytem (1) in the following form:

20 $$\begin{aligned} \begin{aligned}{}&^\textsf{ABC}\mathscr {D}_{t }^{\theta } \textsf{p}= \textsf{F}(x,t,\textsf{p},\textsf{w}),\\&^\textsf{ABC}\mathscr {D}_{t }^{\theta } \textsf{w}= \textsf{G}(x,t,\textsf{p},\textsf{w}). \end{aligned} \end{aligned}$$

Constrained by:

$$\begin{aligned} \begin{aligned}{}&\textsf{p}(x,0)=\textsf{p}_0(x,t),\\&\textsf{w}(x,0)=\textsf{w}_0(x,t). \end{aligned} \end{aligned}$$

apply $\textsf{ABC}$ fractional integral (Definition 2.2) to both sides of the system equations (20),

21 $$\begin{aligned} \begin{aligned}{}&\textsf{p}(x,t) -\textsf{p}(x,0)=\dfrac{1-\theta }{\mathcal {N}(\theta ) } \textsf{K}_1(\textsf{p})+\frac{\theta }{\mathcal {N}(\theta ) \Gamma (\theta )}\int ^{t}_{0} ( t -\textsf{T})^{\theta -1}\,\textsf{K}_1(\textsf{p})\,{\textsf{d}}\textsf{T},\\&\textsf{w}(x,t) -\textsf{w}(x,0)=\dfrac{1-\theta }{\mathcal {N}(\theta ) } \textsf{K}_2(\textsf{w})+\frac{\theta }{\mathcal {N}(\theta ) \Gamma (\theta )}\int ^{t}_{0} ( t -\textsf{T})^{\theta -1}\,\textsf{K}_2(\textsf{w})\,{\textsf{d}}\textsf{T}. \end{aligned} \end{aligned}$$

where $\textsf{K}_1$ and $\textsf{K}_2$ represent the right hand sides of the system, actually, they called the kernels $\textsf{K}_1(x,t,\textsf{p})$ and $\textsf{K}_2(x,t,\textsf{w})$, for simplicity we will write $\textsf{K}_1(\textsf{p})$ and $\textsf{K}_2(\textsf{w})$.

Assume that $\textsf{p}(x,t)$ and $\textsf{w}(x,t)$ have an upper bound if the kernels $\textsf{K}_1(\textsf{p})$ and $\textsf{K}_2(\textsf{w})$ satisfy the Lipschitz condition, hence

22 $$\begin{aligned} \begin{aligned}{}&\parallel \textsf{K}_1(\textsf{p})-\textsf{K}_1(\textsf{p}_1)\parallel \le \varrho _1 \parallel \textsf{p}-\textsf{p}_1 \parallel , \\&\parallel \textsf{K}_2(\textsf{w})-\textsf{K}_2(\textsf{w}_1)\parallel \le \varrho _2 \parallel \textsf{w}-\textsf{w}_1 \parallel . \end{aligned} \end{aligned}$$

The subsequent iterative formulas for $\textsf{p}(x,t)$ and $\textsf{w}(x,t)$ are formulated:

23 $$\begin{aligned} \begin{aligned}{}&\textsf{p}_{\mathfrak {r}+1} =\dfrac{1-\theta }{\mathcal {N}(\theta ) } \textsf{K}_1(\textsf{p}_\mathfrak {r})+\frac{\theta }{\mathcal {N}(\theta ) \Gamma (\theta )}\int ^{t}_{0} ( t -\textsf{T})^{\theta -1}\,\textsf{K}_1(\textsf{p}_\mathfrak {r})\,{\textsf{d}}\textsf{T},\\&\textsf{w}_{\mathfrak {r}+1} =\dfrac{1-\theta }{\mathcal {N}(\theta ) } \textsf{K}_2(\textsf{w}_\mathfrak {r})+\frac{\theta }{\mathcal {N}(\theta ) \Gamma (\theta )}\int ^{t}_{0} ( t -\textsf{T})^{\theta -1}\,\textsf{K}_2(\textsf{w}_\mathfrak {r})\,{\textsf{d}}\textsf{T}. \end{aligned} \end{aligned}$$

The $\textsf{p}$ and $\textsf{w}$ recursive terms will be:

24 $$\begin{aligned} \begin{aligned} \mathcal {V}(x,t)&= \textsf{p}_\mathfrak {r}-\textsf{p}_{\mathfrak {r}-1}\\&=\dfrac{1-\theta }{\mathcal {N}(\theta ) }\bigg ( \textsf{K}_1(\textsf{p}_{\mathfrak {r}-1}) -\textsf{K}_1(\textsf{p}_{\mathfrak {r}-2})\bigg )+\frac{\theta }{\mathcal {N} (\theta ) \Gamma (\theta )}\int ^{t}_{0} ( t -\textsf{T})^{\theta -1}\bigg ( \textsf{K}_1(\textsf{p}_{\mathfrak {r}-1}) -\textsf{K}_1(\textsf{p}_{\mathfrak {r}-2})\bigg ){\textsf{d}}\textsf{T}, \end{aligned} \end{aligned}$$

and

25 $$\begin{aligned} \begin{aligned} \mathcal {W}(x,t)&= \textsf{w}_\mathfrak {r}-\textsf{w}_{\mathfrak {r}-1}\\&=\dfrac{1-\theta }{\mathcal {N}(\theta ) }\bigg ( \textsf{K}_2(\textsf{w}_{\mathfrak {r}-1}) -\textsf{K}_2(\textsf{w}_{\mathfrak {r}-2})\bigg )+\frac{\theta }{\mathcal {N} (\theta ) \Gamma (\theta )}\int ^{t}_{0} ( t -\textsf{T})^{\theta -1} \bigg ( \textsf{K}_2(\textsf{w}_{\mathfrak {r}-1})-\textsf{K}_2(\textsf{w}_{\mathfrak {r}-2})\bigg ) {\textsf{d}}\textsf{T}, \end{aligned} \end{aligned}$$

where the estimate solutions for $\textsf{p}$ and $\textsf{w}$ are:

26 $$\begin{aligned} \textsf{p}_ \mathfrak {r}(x,t)=\sum _{j=0}^{\mathfrak {r}}\mathcal {V}_j(x,t), \end{aligned}$$

and

27 $$\begin{aligned} \textsf{w}_ \mathfrak {r}(x,t)=\sum _{j=0}^{\mathfrak {r}}\mathcal {W}_j(x,t) . \end{aligned}$$

Hence,

28 $$\begin{aligned} \begin{aligned} \parallel \textsf{p}_ \mathfrak {r}(x,t) \parallel&=\parallel \textsf{p}_\mathfrak {r} -\textsf{p}_{\mathfrak {r}-1} \parallel \\&=\parallel \dfrac{1-\theta }{\mathcal {N}(\theta ) }\bigg ( \textsf{K}_1(\textsf{p}_{\mathfrak {r}-1}) -\textsf{K}_1(\textsf{p}_{\mathfrak {r}-2})\bigg )+\frac{\theta }{\mathcal {N}(\theta ) \Gamma (\theta )}\int ^{t}_{0} ( t -\textsf{T})^{\theta -1} \bigg ( \textsf{K}_1(\textsf{p}_{\mathfrak {r}-1}) -\textsf{K}_1(\textsf{p}_{\mathfrak {r}-2})\bigg ){\textsf{d}}\textsf{T}\parallel \\&\le \dfrac{1-\theta }{\mathcal {N}(\theta ) }\parallel \textsf{K}_1(\textsf{p}_{\mathfrak {r}-1}) -\textsf{K}_1(\textsf{p}_{\mathfrak {r}-2})\parallel +\frac{\theta }{\mathcal {N}(\theta ) \Gamma (\theta )}\int ^{t}_{0} ( t -\textsf{T})^{\theta -1}\parallel \textsf{K}_1 (\textsf{p}_{\mathfrak {r}-1})-\textsf{K}_1(\textsf{p}_{\mathfrak {r}-2})\parallel {\textsf{d}}\textsf{T} \\&\le \dfrac{1-\theta }{\mathcal {N}(\theta ) } \varrho _1\parallel \textsf{p}_{\mathfrak {r}-1} -\textsf{p}_{\mathfrak {r}-2}\parallel +\frac{\theta }{\mathcal {N}(\theta ) \Gamma (\theta )} \varrho _1 \int ^{t}_{0} ( t -\textsf{T})^{\theta -1}\parallel \textsf{p}_{\mathfrak {r}-1} -\textsf{p}_{\mathfrak {r}-2}\parallel {\textsf{d}}\textsf{T}, \end{aligned} \end{aligned}$$

similarly,

29 $$\begin{aligned} \begin{aligned} \parallel \textsf{w}_ \mathfrak {r}(x,t) \parallel&=\parallel \textsf{w}_\mathfrak {r} -\textsf{w}_{\mathfrak {r}-1} \parallel \\&\le \dfrac{1-\theta }{\mathcal {N}(\theta ) } \varrho _2\parallel \textsf{w}_{\mathfrak {r}-1} -\textsf{w}_{\mathfrak {r}-2}\parallel +\frac{\theta }{\mathcal {N}(\theta ) \Gamma (\theta )} \varrho _2 \int ^{t}_{0} ( t -\textsf{T})^{\theta -1}\parallel \textsf{w}_{\mathfrak {r}-1} -\textsf{w}_{\mathfrak {r}-2}\parallel {\textsf{d}}\textsf{T}. \end{aligned} \end{aligned}$$

### Theorem 4.1

If the following inequalities are satisfied, then the proposed system (20) having a solution:

30 $$\begin{aligned} \begin{aligned}{}&\dfrac{1-\theta }{\mathcal {N}(\theta ) }\,\,\varrho _1\,\, +\frac{\theta }{\mathcal {N}(\theta ) \Gamma (\theta )} \,\, \varrho _1 \,\, t_0^\theta<1,\\&\dfrac{1-\theta }{\mathcal {N}(\theta ) }\,\, \varrho _2\,\, +\frac{\theta }{\mathcal {N}(\theta ) \Gamma (\theta )} \,\, \varrho _2 \,\, t_0^\theta <1. \end{aligned} \end{aligned}$$

### Proof

From the existence of equation (26) as a solution os the first equation of the proposed system, assume that:

31 $$\begin{aligned} \textsf{p}(x,t)-\textsf{p}(x,0)=\varXi _\mathfrak {r}(x,t), \end{aligned}$$

then

32 $$\begin{aligned} \begin{aligned} \parallel \varXi (x,t) \parallel&=\parallel \dfrac{1-\theta }{\mathcal {N}(\theta ) }\bigg ( \textsf{K}_1(\textsf{p})-\textsf{K}_1(\textsf{p}_{\mathfrak {r}-1})\bigg )+\frac{\theta }{\mathcal {N}(\theta ) \Gamma (\theta )}\int ^{t}_{0} ( t -\textsf{T})^{\theta -1}\bigg ( \textsf{K}_1(\textsf{p})-\textsf{K}_1(\textsf{p}_{\mathfrak {r}-1})\bigg )\parallel \\&\le \dfrac{1-\theta }{\mathcal {N}(\theta ) } \varrho _1\parallel \textsf{p}-\textsf{p}_{\mathfrak {r}-1}\parallel +\frac{\theta }{\mathcal {N}(\theta ) \Gamma (\theta )} \varrho _1 t^\theta \parallel \textsf{p}-\textsf{p}_{\mathfrak {r}-1}\parallel . \end{aligned} \end{aligned}$$

By assuming that $\textsf{p}(x,t)$ is bounded function, apply recursive method for Eq. (28), we get

33 $$\begin{aligned} \parallel \textsf{p}_\mathfrak {r}(x,t) \parallel =\bigg \{\dfrac{1-\theta }{\mathcal {N}(\theta ) } \varrho _1+ \frac{\theta }{\mathcal {N}(\theta ) \Gamma (\theta )} \varrho _1 t^\theta \bigg \}^\mathfrak {r}. \end{aligned}$$

Using relation (33) with the inequality (32), we obtain:

34 $$\begin{aligned} \parallel \varXi _\mathfrak {r}(x,t) \parallel \le \bigg \{\dfrac{1-\theta }{\mathcal {N}(\theta ) } + \frac{\theta }{\mathcal {N}(\theta ) \Gamma (\theta )} t^\theta \bigg \}^{\mathfrak {r}+1} \varrho _1^{\mathfrak {r}+1}. \end{aligned}$$

At $t=t_0$, Eq. (34) becomes:

35 $$\begin{aligned} \parallel \varXi _\mathfrak {r}(x,t) \parallel \le \bigg \{\dfrac{1-\theta }{\mathcal {N}(\theta ) } + \frac{\theta }{\mathcal {N}(\theta ) \Gamma (\theta )} t_0^\theta \bigg \}^{\mathfrak {r}+1} \varrho _1^{\mathfrak {r}+1}. \end{aligned}$$

Similarily, we can show that,

36 $$\begin{aligned} \parallel \textsf{H}_\mathfrak {r}(x,t) \parallel \le \bigg \{\dfrac{1-\theta }{\mathcal {N}(\theta ) } + \frac{\theta }{\mathcal {N}(\theta ) \Gamma (\theta )} t_0^\theta \bigg \}^{\mathfrak {r}+1} \varrho _2^{\mathfrak {r}+1}, \end{aligned}$$

where $\textsf{H}_\mathfrak {r}(x,t)= \textsf{w}(x,t)-\textsf{w}(x,0)$. To prove the uniqueness of the solution, consider that $\textsf{p}(x,t)$ has two solutions $\textsf{p}(x,t)$ and $\textsf{q}(x,t)$, hence

37 $$\begin{aligned} \textsf{p}(x,t) - \textsf{q}(x,t)=\dfrac{1-\theta }{\mathcal {N}(\theta ) }\bigg ( \textsf{K}_1(\textsf{p})-\textsf{K}_1(\textsf{q})\bigg )+\frac{\theta }{\mathcal {N}(\theta ) \Gamma (\theta )}\int ^{t}_{0} ( t -\textsf{T})^{\theta -1}\bigg ( \textsf{K}_1(\textsf{p})-\textsf{K}_1(\textsf{q})\bigg ){\textsf{d}}\textsf{T}. \end{aligned}$$

Putting norm on both sides of Eq. (37),

38 $$\begin{aligned} \begin{aligned} \parallel \textsf{p}(x,t) - \textsf{q}(x,t)\parallel&=\parallel \dfrac{1-\theta }{\mathcal {N}(\theta ) }\bigg ( \textsf{K}_1(\textsf{p})-\textsf{K}_1(\textsf{q})\bigg )+\frac{\theta }{\mathcal {N}(\theta ) \Gamma (\theta )}\int ^{t}_{0} ( t -\textsf{T})^{\theta -1}\bigg ( \textsf{K}_1(\textsf{p})-\textsf{K}_1(\textsf{q})\bigg ){\textsf{d}}\textsf{T}\parallel \\&\le \dfrac{1-\theta }{\mathcal {N}(\theta ) }\parallel \textsf{K}_1(\textsf{p})-\textsf{K}_1(\textsf{q})\parallel +\frac{\theta }{\mathcal {N}(\theta ) \Gamma (\theta )}\int ^{t}_{0} ( t -\textsf{T})^{\theta -1}\parallel \textsf{K}_1(\textsf{p})-\textsf{K}_1(\textsf{q})\parallel {\textsf{d}}\textsf{T}\\&\le \dfrac{1-\theta }{\mathcal {N}(\theta ) }\varrho _1 \parallel \textsf{p}(x,t) - \textsf{q}(x,t)\parallel + \frac{\theta }{\mathcal {N}(\theta ) \Gamma (\theta )}\varrho _1 t^\theta \parallel \textsf{p}(x,t) - \textsf{q}(x,t)\parallel . \end{aligned} \end{aligned}$$

Thus,

39 $$\begin{aligned} \parallel \textsf{p}(x,t) - \textsf{q}(x,t)\parallel \bigg ( 1-\dfrac{1-\theta }{\mathcal {N}(\theta ) }\varrho _1- \frac{\theta }{\mathcal {N}(\theta ) \Gamma (\theta )}\varrho _1 t^\theta \bigg )\le 0. \end{aligned}$$

Hence, $\parallel \textsf{p}(x,t) - \textsf{q}(x,t)\parallel =0$ when $\bigg ( 1-\dfrac{1-\theta }{\mathcal {N}(\theta ) }\varrho _1- \frac{\theta }{\mathcal {N}(\theta ) \Gamma (\theta )}\varrho _1 t^\theta \bigg )>0$, therefore $\textsf{p}(x,t)=\textsf{q}(x,t)$.

The same conclusion can be drawn for $\textsf{w}(x,t)$. $\square$

## Solution of the TFBZS using DLM

In this section, the DLM is applied to the TFBZS to find approximate solutions, the system will be investigated under two types of initial conditions^22^.

**Case I : For** $\beta =\gamma =0$ , Eq. (1) will be:

40 $$\begin{aligned} \begin{aligned}{}&\frac{\partial ^\theta \textsf{p} }{\partial t^\theta }= \frac{\partial ^{2} \textsf{p} }{\partial x^{2}}+\textsf{p}-\textsf{p}^2 -\delta \textsf{p} \textsf{w},\\&\frac{\partial ^\theta \textsf{w} }{\partial t^\theta }= \frac{\partial ^{2} \textsf{w} }{\partial x^{2}} -\lambda \textsf{p} \textsf{w}. \end{aligned} \end{aligned}$$

Under initial conditions:

$$\begin{aligned} \begin{aligned}{}&\textsf{p}(x,0)=\frac{1}{\left( \exp \left( \sqrt{\frac{\lambda }{6}} x\right) +1\right) ^2},\\&\textsf{w}(x,0)=\frac{(1-\lambda ) \exp \left( \sqrt{\frac{\lambda }{6}} x\right) \left( \exp \left( \sqrt{\frac{\lambda }{6}} x\right) +2\right) }{\delta \left( \exp \left( \sqrt{\frac{\lambda }{6}} x\right) +1\right) ^2}. \end{aligned} \end{aligned}$$

The exact solution at $\theta =1$ is:

$$\begin{aligned} \begin{aligned}{}&\textsf{p}(x,t)=\frac{\exp \bigg (\frac{5 \lambda }{3} t\bigg )}{\left( \exp \bigg ( \frac{5 \lambda }{6} t\bigg )+\exp \left( \sqrt{\frac{\lambda }{6}} x\right) \right) ^2},\\&\textsf{w}(x,t)=\frac{(1-\lambda ) \exp \left( \sqrt{\frac{\lambda }{6}} x\right) \left( 2 \exp \bigg (\frac{5 \lambda }{6} ) t\bigg )+\exp \left( \sqrt{\frac{\lambda }{6}} x\right) \right) }{\delta \left( \exp \bigg (\frac{5 \lambda }{6} t\bigg )+\exp \left( \sqrt{\frac{\lambda }{6}} x\right) \right) ^2}. \end{aligned} \end{aligned}$$

**Using Caputo DLTM**:

Apply the DL formula (17) into both sides of the system (40),

41 $$\begin{aligned} \begin{aligned}{}&\mathscr {L}_x \mathscr {L}_t \bigg \{\ ^\textsf{C}\mathscr {D}_{t }^{\theta } \textsf{p}\bigg \} =\mathscr {L}_x \mathscr {L}_t \bigg \{\ \frac{\partial ^2 \textsf{p} }{\partial x^2} +\textsf{p} -\textsf{p}^2-\delta \textsf{p} \textsf{w}\bigg \},\\&\mathscr {L}_x \mathscr {L}_t \bigg \{\ ^\textsf{C}\mathscr {D}_{t }^{\theta } \textsf{w}\bigg \} =\mathscr {L}_x \mathscr {L}_t \bigg \{\ \frac{\partial ^2 \textsf{w} }{\partial x^2} -\lambda \textsf{p} \textsf{w}\bigg \}. \end{aligned} \end{aligned}$$

Then

42 $$\begin{aligned} S ^\theta \mathscr {L}_x \mathscr {L}_t \bigg \{\ \textsf{p}\bigg \}- S ^{\theta -1} \mathscr {L}_x \textsf{p}(x,0) =\mathscr {L}_x \mathscr {L}_t \bigg \{\ \frac{\partial ^2 \textsf{p} }{\partial x^2}\bigg \}+\mathscr {L}_x \mathscr {L}_t \bigg \{\ \textsf{p} \bigg \}-\mathscr {L}_x \mathscr {L}_t \bigg \{\ \mathscr {N}_\mathfrak {j} \bigg \}-\delta \mathscr {L}_x \mathscr {L}_t \bigg \{\ \mathscr {M}_\mathfrak {j} \bigg \}, \end{aligned}$$

and

43 $$\begin{aligned} S ^\theta \mathscr {L}_x \mathscr {L}_t \bigg \{\ \textsf{w}\bigg \}- S ^{\theta -1} \mathscr {L}_x \textsf{w}(x,0) =\mathscr {L}_x \mathscr {L}_t \bigg \{\ \frac{\partial ^2 \textsf{w} }{\partial x^2}\bigg \}-\lambda \mathscr {L}_x \mathscr {L}_t \bigg \{\ \mathscr {M}_\mathfrak {j} \bigg \}. \end{aligned}$$

where, $\mathscr {N}_\mathfrak {j}$ and $\mathscr {M}_\mathfrak {j}$ are the Adomian polynomials for the nonlinear terms $\textsf{p}^2$ and $\textsf{p}\textsf{w}$ respectively. Hence,

44 $$\begin{aligned} \begin{aligned}{}&\mathscr {L}_x \mathscr {L}_t \bigg \{\ \textsf{p}\bigg \}=\frac{1}{ S } \mathscr {L}_x \textsf{p}(x,0) +\frac{1}{ S ^\theta }\bigg [\mathscr {L}_x \mathscr {L}_t \bigg \{\ \frac{\partial ^2 \textsf{p} }{\partial x^2}\bigg \}+\mathscr {L}_x \mathscr {L}_t \bigg \{\ \textsf{p} \bigg \}-\mathscr {L}_x \mathscr {L}_t \bigg \{\ \mathscr {N}_\mathfrak {j} \bigg \}-\delta \mathscr {L}_x \mathscr {L}_t \bigg \{\ \mathscr {M}_\mathfrak {j} \bigg \}\bigg ],\\&\mathscr {L}_x \mathscr {L}_t \bigg \{\ \textsf{w}\bigg \}=\frac{1}{ S } \mathscr {L}_x \textsf{w}(x,0) +\frac{1}{ S ^\theta }\bigg [\mathscr {L}_x \mathscr {L}_t \bigg \{\ \frac{\partial ^2 \textsf{w} }{\partial x^2}\bigg \}-\lambda \mathscr {L}_x \mathscr {L}_t \bigg \{\ \mathscr {M}_\mathfrak {j} \bigg \}\bigg ]. \end{aligned} \end{aligned}$$

To find the unknown functions $\textsf{p}$ and $\textsf{w}$, take inverse DL to both sides of Eq. (44), hence

45 $$\begin{aligned} \textsf{p}_0&=\textsf{p}(x,0)=\frac{1}{\left( \exp \left( \sqrt{\frac{\lambda }{6}} x\right) +1\right) ^2}, \end{aligned}$$

46 $$\begin{aligned} \textsf{p}_1&=\mathscr {L}^{-1}_x \mathscr {L}^{-1}_t \frac{1}{ S ^\theta }\bigg [ \mathscr {L}_x \mathscr {L}_t \bigg \{\ \frac{\partial ^2 \textsf{p}_0 }{\partial x^2}+ \textsf{p}_0 - \mathscr {N}_0 -\delta \mathscr {M}_0 \bigg \}\bigg ]\nonumber \\&=\frac{5 \lambda t^{\theta } e^{\frac{\sqrt{\lambda } x}{\sqrt{6}}}}{3 \Gamma (\theta +1) \left( e^{\frac{\sqrt{\lambda } x}{\sqrt{6}}}+1\right) ^3}, \end{aligned}$$

47 $$\begin{aligned} \textsf{p}_2&=\mathscr {L}^{-1}_x \mathscr {L}^{-1}_t \frac{1}{ S ^\theta }\bigg [ \mathscr {L}_x \mathscr {L}_t \bigg \{\ \frac{\partial ^2 \textsf{p}_1 }{\partial x^2}+ \textsf{p}_1 - \mathscr {N}_1 -\delta \mathscr {M}_1 \bigg \}\bigg ]\nonumber \\&=\frac{25 \lambda ^2 t^{2 \theta } e^{\frac{\sqrt{\lambda } x}{\sqrt{6}}} \left( 2 e^{\frac{\sqrt{\lambda } x}{\sqrt{6}}}-1\right) }{18 \Gamma (2 \theta +1) \left( e^{\frac{\sqrt{\lambda } x}{\sqrt{6}}}+1\right) ^4},\nonumber \\&:\end{aligned}$$

Similarily,

48 $$\begin{aligned} \textsf{w}_0&=\textsf{w}(x,0)=\frac{(1-\lambda ) \exp \left( \sqrt{\frac{\lambda }{6}} x\right) \left( \exp \left( \sqrt{\frac{\lambda }{6}} x\right) +2\right) }{\delta \left( \exp \left( \sqrt{\frac{\lambda }{6}} x\right) +1\right) ^2}, \end{aligned}$$

49 $$\begin{aligned} \textsf{w}_1&=\mathscr {L}^{-1}_x \mathscr {L}^{-1}_t \frac{1}{ S ^\theta }\bigg [ \mathscr {L}_x \mathscr {L}_t \bigg \{\ \frac{\partial ^2 \textsf{w}_0 }{\partial x^2}-\lambda \mathscr {M}_0 \bigg \} \bigg ]\nonumber \\&=\frac{5 (\lambda -1) \lambda t^{\theta } e^{\frac{\sqrt{\lambda } x}{\sqrt{6}}}}{3 \delta \Gamma (\theta +1) \left( e^{\frac{\sqrt{\lambda } x}{\sqrt{6}}}+1\right) ^3}, \end{aligned}$$

50 $$\begin{aligned} \textsf{w}_2&=\mathscr {L}^{-1}_x \mathscr {L}^{-1}_t \frac{1}{ S ^\theta }\bigg [ \mathscr {L}_x \mathscr {L}_t \bigg \{\ \frac{\partial ^2 \textsf{w}_1 }{\partial x^2}-\lambda \mathscr {M}_1 \bigg \} \bigg ]\nonumber \\&=\frac{25 (\lambda -1) \lambda ^2 t^{2 \theta } e^{\frac{\sqrt{\lambda } x}{\sqrt{6}}} \left( 2 e^{\frac{\sqrt{\lambda } x}{\sqrt{6}}}-1\right) }{18 \delta \Gamma (2 \theta +1) \left( e^{\frac{\sqrt{\lambda } x}{\sqrt{6}}}+1\right) ^4},\nonumber \\&:\end{aligned}$$

If we truncate the solution at two iterations, the form of the approximate series solution will ultimately be as follows:

51 $$\begin{aligned} \begin{aligned}{}&{}^\textsf{C}_1\textsf{p}(x,t)=\sum _{\mathfrak {j}=0}^{2} \textsf{p}_\mathfrak {j},\\&{}^\textsf{C}_1\textsf{w}(x,t)=\sum _{\mathfrak {j}=0}^{2} \textsf{w}_\mathfrak {j}. \end{aligned} \end{aligned}$$

Table 1 represents the exact and approximate values of the unknown functions $\textsf{p}$ and $\textsf{w}$ and the absolute error for case I under Caputo fractional derivative at $\theta =1$, $t=0.01$, $\delta =1$ and $\lambda =1.5$ or varying *x* values.

**Table 1:** Precise and estimated solutions for the TFBZS at $\theta =1$, $t=0.01$, $\delta =1$ and $\lambda =1.5$.

*x*	$\textsf{p}(x,t)$			$\textsf{w}(x,t)$		
	Precise solution	Estimated solution	Abs. error	Precise solution	Estimated solution	Abs. error
− 10	0.986823	0.986823	4.03097 E− 9	0.0065884	0.0065884	2.01549 E− 9
− 6	0.908468	0.908468	1.68005 E− 8	0.0457662	0.0457662	8.40027 E− 9
− 2	0.538036	0.538036	5.15251 E− 8	0.230982	0.230982	2.57626 E− 8
2	0.0736613	0.0736613	2.86303 E− 8	0.463169	0.463169	1.43151 E− 8
6	0.0023034	0.0023034	4.64909 E− 9	0.498848	0.498848	2.32455 E− 9
10	0.0000459	0.0000459	1.13672 E− 10	0.499977	0.499977	5.68359 E− 11

**Using Atangana-Baleanu-Caputo DLTM**:

Apply formula (19) to the TFBZS (40),

52 $$\begin{aligned} \begin{aligned}{}&\mathscr {L}_x \mathscr {L}_t \bigg \{\ ^\textsf{ABC}\mathscr {D}_{t }^{\theta } \textsf{p}\bigg \} =\mathscr {L}_x \mathscr {L}_t \bigg \{\ \frac{\partial ^2 \textsf{p} }{\partial x^2} +\textsf{p} -\textsf{p}^2-\delta \textsf{p} \textsf{w}\bigg \},\\&\mathscr {L}_x \mathscr {L}_t \bigg \{\ ^\textsf{ABC}\mathscr {D}_{t }^{\theta } \textsf{w}\bigg \} =\mathscr {L}_x \mathscr {L}_t \bigg \{\ \frac{\partial ^2 \textsf{w} }{\partial x^2} -\lambda \textsf{p} \textsf{w}\bigg \}. \end{aligned} \end{aligned}$$

Then

53 $$\begin{aligned} \begin{aligned}{}&\frac{ S ^{\theta }}{(1-\theta ) \left( S ^\theta +\frac{\theta }{1-\theta }\right) } \mathscr {L}_x \mathscr {L}_t \bigg \{\ \textsf{p}\bigg \}-\frac{ S ^{\theta -1 }}{(1-\theta )\left( S ^\theta +\frac{\theta }{1-\theta }\right) } \mathscr {L}_x \textsf{p}(x,0) =\mathscr {L}_x \mathscr {L}_t \bigg \{\ \frac{\partial ^2 \textsf{p} }{\partial x^2}\bigg \}+\mathscr {L}_x \mathscr {L}_t \bigg \{\ \textsf{p} \bigg \}\\&\quad -\mathscr {L}_x \mathscr {L}_t \bigg \{\ \mathscr {N}_\mathfrak {j} \bigg \}-\delta \mathscr {L}_x \mathscr {L}_t \bigg \{\ \mathscr {M}_\mathfrak {j} \bigg \}, \end{aligned} \end{aligned}$$

and,

54 $$\begin{aligned} \frac{ S ^{\theta }}{(1-\theta ) \left( S ^\theta +\frac{\theta }{1-\theta }\right) } \mathscr {L}_x \mathscr {L}_t \bigg \{\ \textsf{w}\bigg \}-\frac{ S ^{\theta -1 }}{(1-\theta ) \left( S ^\theta +\frac{\theta }{1-\theta }\right) } \mathscr {L}_x \textsf{w}(x,0) =\mathscr {L}_x \mathscr {L}_t \bigg \{\ \frac{\partial ^2 \textsf{w} }{\partial x^2}\bigg \}-\lambda \mathscr {L}_x \mathscr {L}_t \bigg \{\ \mathscr {M}_\mathfrak {j} \bigg \}. \end{aligned}$$

Hence,

55 $$\begin{aligned} \begin{aligned} \mathscr {L}_x \mathscr {L}_t \bigg \{\ \textsf{p}\bigg \}&=\frac{1}{ S } \mathscr {L}_x \textsf{p}(x,0) +\bigg (1-\theta +\dfrac{\theta }{ S ^\theta } \bigg )\bigg [\mathscr {L}_x \mathscr {L}_t \bigg \{\ \frac{\partial ^2 \textsf{p} }{\partial x^2}\bigg \}+\mathscr {L}_x \mathscr {L}_t \bigg \{\ \textsf{p} \bigg \}\\&-\mathscr {L}_x \mathscr {L}_t \bigg \{\ \mathscr {N}_\mathfrak {j} \bigg \}-\delta \mathscr {L}_x \mathscr {L}_t \bigg \{\ \mathscr {M}_\mathfrak {j} \bigg \}\bigg ], \\ \mathscr {L}_x \mathscr {L}_t \bigg \{\ \textsf{w}\bigg \}&=\frac{1}{ S } \mathscr {L}_x \textsf{w}(x,0) +\bigg (1-\theta +\dfrac{\theta }{ S ^\theta } \bigg )\bigg [\mathscr {L}_x \mathscr {L}_t \bigg \{\ \frac{\partial ^2 \textsf{w} }{\partial x^2}\bigg \}-\lambda \mathscr {L}_x \mathscr {L}_t \bigg \{\ \mathscr {M}_\mathfrak {j} \bigg \}\bigg ]. \end{aligned} \end{aligned}$$

Taking inverse DL to both sides of equation (55) to obtain the recursive values of $\textsf{p}$ and $\textsf{w}$.

56 $$\begin{aligned} \textsf{p}_0&=\textsf{p}(x,0)=\frac{1}{\left( \exp \left( \sqrt{\frac{\lambda }{6}} x\right) +1\right) ^2}, \end{aligned}$$

57 $$\begin{aligned} \textsf{p}_1&=\mathscr {L}^{-1}_x \mathscr {L}^{-1}_t \bigg (1-\theta +\dfrac{\theta }{ S ^\theta } \bigg )\bigg [ \mathscr {L}_x \mathscr {L}_t \bigg \{\ \frac{\partial ^2 \textsf{p}_0 }{\partial x^2}+ \textsf{p}_0 - \mathscr {N}_0 -\delta \mathscr {M}_0 \bigg \}\bigg ]\nonumber \\&=\frac{5 \lambda e^{\frac{\sqrt{\lambda } x}{\sqrt{6}}} \left( -\theta +\frac{\theta t^{\theta }}{\Gamma (\theta +1)}+1\right) }{3 \left( e^{\frac{\sqrt{\lambda } x}{\sqrt{6}}}+1\right) ^3}, \end{aligned}$$

58 $$\begin{aligned} \textsf{p}_2&=\mathscr {L}^{-1}_x \mathscr {L}^{-1}_t \bigg (1-\theta +\dfrac{\theta }{ S ^\theta } \bigg )\bigg [ \mathscr {L}_x \mathscr {L}_t \bigg \{\ \frac{\partial ^2 \textsf{p}_1 }{\partial x^2}+ \textsf{p}_1 - \mathscr {N}_1 -\delta \mathscr {M}_1 \bigg \}\bigg ]\nonumber \\&=\frac{25 \lambda ^2 e^{\frac{\sqrt{\lambda } x}{\sqrt{6}}} \left( 2 e^{\frac{\sqrt{\lambda } x}{\sqrt{6}}}-1\right) \left( \Gamma (\theta +1) \left( (\theta -1)^2 \Gamma (2 \theta +1)+\theta ^2 t^{2 \theta }\right) -2 (\theta -1) \theta \Gamma (2 \theta +1) t^{\theta }\right) }{18 \Gamma (\theta +1) \Gamma (2 \theta +1) \left( e^{\frac{\sqrt{\lambda } x}{\sqrt{6}}}+1\right) ^4},\nonumber \\&:\end{aligned}$$

Similarily,

59 $$\begin{aligned} \textsf{w}_0&=\textsf{w}(x,0)=\frac{(1-\lambda ) \exp \left( \sqrt{\frac{\lambda }{6}} x\right) \left( \exp \left( \sqrt{\frac{\lambda }{6}} x\right) +2\right) }{\delta \left( \exp \left( \sqrt{\frac{\lambda }{6}} x\right) +1\right) ^2}, \end{aligned}$$

60 $$\begin{aligned} \textsf{w}_1&=\mathscr {L}^{-1}_x \mathscr {L}^{-1}_t \bigg (1-\theta +\dfrac{\theta }{ S ^\theta } \bigg )\bigg [ \mathscr {L}_x \mathscr {L}_t \bigg \{\ \frac{\partial ^2 \textsf{w}_0 }{\partial x^2}-\lambda \mathscr {M}_0 \bigg \} \bigg ]\nonumber \\&=\frac{5 (\lambda -1) \lambda e^{\frac{\sqrt{\lambda } x}{\sqrt{6}}} \left( -\theta +\frac{\theta t^{\theta }}{\Gamma (\theta +1)}+1\right) }{3 \delta \left( e^{\frac{\sqrt{\lambda } x}{\sqrt{6}}}+1\right) ^3}, \end{aligned}$$

61 $$\begin{aligned} \textsf{w}_2&=\mathscr {L}^{-1}_x \mathscr {L}^{-1}_t \bigg (1-\theta +\dfrac{\theta }{ S ^\theta } \bigg )\bigg [ \mathscr {L}_x \mathscr {L}_t \bigg \{\ \frac{\partial ^2 \textsf{w}_1 }{\partial x^2}-\lambda \mathscr {M}_1 \bigg \} \bigg ]\nonumber \\&=\frac{1}{3 \delta \Gamma (\alpha +1) \Gamma (2 \alpha +1) \left( e^{\frac{\sqrt{\lambda } x}{\sqrt{6}}}+1\right) ^5}\bigg [5 e^{\frac{\sqrt{\lambda } x}{\sqrt{6}}} \left( e^{\sqrt{\frac{2}{3}} \sqrt{\lambda } x}+2 e^{\frac{\sqrt{\lambda } x}{\sqrt{6}}}-1\right) \nonumber \\&(\lambda -1) \lambda ^2 \left( \Gamma (\alpha +1) \left( (\alpha -1)^2 \Gamma (2 \alpha +1)+\alpha ^2 t^{2 \alpha }\right) -2 (\alpha -1) \alpha \Gamma (2 \alpha +1) t^{\alpha }\right) \bigg ],\nonumber \\&:\end{aligned}$$

The final form of the estimated series solution with two iterations will ultimately be as follows:

62 $$\begin{aligned} {}^\textsf{ABC}\textsf{p}(x,t)=\sum _{\mathfrak {j}=0}^{2} \textsf{p}_\mathfrak {j},\,\,\,\,\,\,\,\,\,\,\,\,\,\,\,\,\,\,\,\,\,\, {}^\textsf{ABC}\textsf{w}(x,t)=\sum _{\mathfrak {j}=0}^{2} \textsf{w}_\mathfrak {j}. \end{aligned}$$

Table 2 represents the Precise and estimated solutions for the unknown functions $\textsf{p}$ and $\textsf{w}$ of the TFBZS and the absolute error for case I under $\textsf{ABC}$ fractional derivative at $\theta =1$, $t=0.01$, $\delta =1$ and $\lambda =1.5$ for various values of *x*.

**Table 2:** Precise and estimated solutions for the TFBZS at $\theta =1$, $t=0.01$, $\delta =1$ and $\lambda =1.5$.

*x*	$\textsf{p}(x,t)$			$\textsf{w}(x,t)$		
	Precise solution	Estimated solution	Abs. error	Precise solution	Estimated solution	Abs. error
− 10	0.986823	0.986823	4.03097 E−9	0.0065884	0.0065885	9.90365 E−8
− 6	0.908468	0.908468	1.68005 E−8	0.0457662	0.0457666	4.11951 E−7
− 2	0.538036	0.538036	5.15251 E−8	0.230982	0.230981	1.26664 E−6
2	0.0736613	0.0736613	2.86303 E−8	0.463169	0.463170	7.03231 E−7
6	0.0023034	0.0023034	4.64909 E−9	0.498848	0.498848	1.13322 E−7
10	0.0000459	0.0000459	1.13672 E−10	0.499977	0.499977	2.76832 E−9

**Case II : For** $\gamma =\lambda$, **and** $\beta =1$, Eq. (1) will be:

63 $$\begin{aligned} \begin{aligned}{}&\frac{\partial ^\theta \textsf{p} }{\partial t^\theta }= \frac{\partial ^{2} \textsf{p} }{\partial x^{2}}+\delta \textsf{w}+\textsf{p}-\textsf{p}^2 -\delta \textsf{p} \textsf{w},\\&\frac{\partial ^\theta \textsf{w} }{\partial t^\theta }= \frac{\partial ^{2} \textsf{w} }{\partial x^{2}}+\lambda \textsf{w} -\lambda \textsf{p} \textsf{w}. \end{aligned} \end{aligned}$$

Under initial conditions conditions:

$$\begin{aligned} \begin{aligned}{}&\textsf{p}(x,0)=\frac{1}{4} \left( \tanh \left( \sqrt{\frac{\lambda }{24}} x\right) -1\right) ^2,\\&\textsf{w}(x,0)=\frac{(\lambda -1)}{4\delta }\left( \tanh \left( \sqrt{\frac{\lambda }{24}} x\right) -1\right) ^2. \end{aligned} \end{aligned}$$

The exact solution at $\theta =1$ is:

$$\begin{aligned} \begin{aligned}{}&\textsf{p}(x,t)=\frac{1}{4} \left( \tanh \left( \sqrt{\frac{\lambda }{24}} x-\frac{1}{12} (5 \lambda ) t\right) -1\right) ^2,\\&\textsf{w}(x,t)=\frac{(\lambda -1)}{4\delta }\left( \tanh \left( \sqrt{\frac{\lambda }{24}} x-\frac{1}{12} (5 \lambda ) t\right) -1\right) ^2. \end{aligned} \end{aligned}$$

**Using Caputo DLTM**:

Apply the DL formula (17) into both sides of the system (63), and follow the same procedure discussed in Case I, we obtain the following solutions:

64 $$\begin{aligned} \textsf{p}_0&=\textsf{p}(x,0),\nonumber \\ \textsf{p}_1&=-\frac{5 \lambda t^{\theta } \left( \tanh \left( \frac{\sqrt{\lambda } x}{2 \sqrt{6}}\right) -1\right) \text {sech}^2\left( \frac{\sqrt{\lambda } x}{2 \sqrt{6}}\right) }{24 \Gamma (\theta +1)}, \end{aligned}$$

65 $$\begin{aligned} \textsf{p}_2&=\frac{1}{5308416 \Gamma (\theta +1)^3 \Gamma (3 \theta +1) \Gamma (4 \theta +1)}\bigg (25 \lambda ^3 t^{3 \theta } \text {sech}^{15}\left( \frac{\sqrt{\lambda } x}{2 \sqrt{6}}\right) \nonumber \\&\left( \cosh \left( \frac{\sqrt{\lambda } x}{\sqrt{6}}\right) -\sinh \left( \frac{\sqrt{\lambda } x}{\sqrt{6}}\right) \right) \left( 3 \sinh \left( \frac{\sqrt{\lambda } x}{\sqrt{6}}\right) +5 \cosh \left( \frac{\sqrt{\lambda } x}{\sqrt{6}}\right) -7\right) \nonumber \\&(5 \lambda \Gamma (3 \theta +1)^2 t^{\theta }+384 (\lambda -1) \Gamma (\theta +1) \Gamma (2\theta +1) \Gamma (4 \theta +1) \cosh ^7\left( \frac{\sqrt{\lambda } x}{2 \sqrt{6}}\right) \nonumber \\&\cosh \left( \frac{1}{2} \sqrt{\frac{3}{2}} \sqrt{\lambda } x\right) -\sinh \left( \frac{1}{2} \sqrt{\frac{3}{2}} \sqrt{\lambda } x\right) \nonumber \\&\left( \lambda ^2-2 \lambda -3\right) \cosh \left( \frac{\sqrt{\lambda } x}{\sqrt{6}}\right) +(\lambda -1)^2 \left( -\sinh \left( \frac{\sqrt{\lambda } x}{\sqrt{6}}\right) \right) -4) \bigg ),\nonumber \\&:\end{aligned}$$

Similarily,

66 $$\begin{aligned} \begin{aligned} \textsf{w}_0&=\textsf{w}(x,0),\\ \textsf{w}_1&=-\frac{5 (\lambda -1) \lambda t^{\theta } \left( \tanh \left( \frac{\sqrt{\lambda } x}{2 \sqrt{6}}\right) -1\right) \text {sech}^2\left( \frac{\sqrt{\lambda } x}{2 \sqrt{6}}\right) }{24 \delta \Gamma (\theta +1)}\\ \textsf{w}_2&=-\frac{1}{9216 \delta ^2 \Gamma (\theta +1)^2 \Gamma (2 \theta +1) \Gamma (3 \theta +1)}\bigg ( 5 (\lambda -1) \lambda ^2 t^{2 \theta } \text {sech}^4\left( \frac{\sqrt{\lambda } x}{2 \sqrt{6}}\right) \\&(16 \delta \Gamma (\theta +1)^2 \Gamma (3 \theta +1) \left( 3 \sinh \left( \frac{\sqrt{\lambda } x}{\sqrt{6}}\right) +17 \cosh \left( \frac{\sqrt{\lambda } x}{\sqrt{6}}\right) +5\right) \\&-5 (\lambda -1) \lambda ^2 \Gamma (2 \theta +1)^2 t^{\theta } \left( \tanh \left( \frac{\sqrt{\lambda } x}{2 \sqrt{6}}\right) -1\right) ^5)\tanh \left( \frac{\sqrt{\lambda } x}{2 \sqrt{6}}\right) -1 \bigg ),\\&:\end{aligned} \end{aligned}$$

Finally, the approximate two iterations series solution of case II under Caputo fractional derivative will be in the form:

67 $$\begin{aligned} \begin{aligned}{}&^\textsf{C}_2\textsf{p}(x,t)=\sum _{\mathfrak {j}=0}^{2} \textsf{p}_\mathfrak {j},\\&^\textsf{C}_2\textsf{w}(x,t)=\sum _{\mathfrak {j}=0}^{2} \textsf{w}_\mathfrak {j}. \end{aligned} \end{aligned}$$

Table 3 represents the exact, approximate and the absolute error results from solving the TFBZS (case II) at $\theta =1$, $t=0.01$, $\delta =2$ and $\lambda =2$ for varying *x*-values.

**Table 3:** Exact and approximate solutions of the TFBZS (case II) at $\theta =1$, $t=0.01$, $\delta =2$ and $\lambda =2$.

*x*	$\textsf{p}(x,t)$			$\textsf{w}(x,t)$		
	Exact	Approx.	Abs. error	Exact	Approx.	Abs. error
− 10	0.993913	0.993914	8.43019 E−7	0.496957	0.496958	1.01806 E−6
− 6	0.941163	0.941170	7.17341 E−6	0.470582	0.470590	8.93003 E−6
− 2	0.582767	0.582778	1.09066 E−5	0.291384	0.291410	2.67739 E−5
2	0.0588943	0.0588787	1.56091 E−5	0.0294472	0.029448	7.98941 E−7
6	0.0009514	0.0009509	4.78581 E−7	0.0004757	0.0004757	2.19483 E−9
10	9.929 E−6	9.924 E−6	5.35382 E−9	4.964 E−6	4.964 E−6	2.94228 E−11

**Using Atangana-Baleanu-Caputo DLTM**:

Apply the DL formula (19) into both sides of the system (63), and perform the same steps presented in Case I to obtain the following results:

68 $$\begin{aligned} \textsf{p}_0&=\textsf{p}(x,0),\nonumber \\ \textsf{p}_1&=\frac{1}{24} (-5) \lambda \left( -\theta +\frac{\theta t^{\theta }}{\Gamma (\theta +1)}+1\right) \left( \tanh \left( \frac{\sqrt{\lambda } x}{2 \sqrt{6}}\right) -1\right) \text {sech}^2\left( \frac{\sqrt{\lambda } x}{2 \sqrt{6}}\right) , \end{aligned}$$

69 $$\begin{aligned} \textsf{p}_2&=\frac{25 \lambda ^3 \text {sech}^{15}\left( \frac{\sqrt{\lambda } x}{2 \sqrt{6}}\right) \left( \cosh \left( \frac{\sqrt{\lambda } x}{\sqrt{6}}\right) -\sinh \left( \frac{\sqrt{\lambda } x}{\sqrt{6}}\right) \right) \left( 3 \sinh \left( \frac{\sqrt{\lambda } x}{\sqrt{6}}\right) +5 \cosh \left( \frac{\sqrt{\lambda } x}{\sqrt{6}}\right) -7\right) }{5308416 \Gamma (\theta +1)^3}\nonumber \\&\quad \bigg (20 \theta ^4 \lambda t^{3\theta } \cosh \left( \frac{1}{2} \sqrt{\frac{3}{2}} \sqrt{\lambda } x\right) -15 \theta ^3 \lambda t^{3 \theta } \cosh \left( \frac{1}{2} \sqrt{\frac{3}{2}} \sqrt{\lambda } x\right) \cosh \left( \frac{\sqrt{\lambda } x}{\sqrt{6}}\right) \nonumber \\&\quad -20 \theta ^3 \lambda t^{3 \theta } \cosh \left( \frac{1}{2} \sqrt{\frac{3}{2}} \sqrt{\lambda } x\right) +15 \theta ^4 \lambda t^{3 \theta } \cosh \left( \frac{1}{2} \sqrt{\frac{3}{2}} \sqrt{\lambda } x\right) \cosh \left( \frac{\sqrt{\lambda } x}{\sqrt{6}}\right) \nonumber \\&\quad 10 \theta ^4 \lambda ^2 t^{3 \theta } \cosh \left( \frac{1}{2} \sqrt{\frac{3}{2}} \sqrt{\lambda } x\right) \cosh \left( \frac{\sqrt{\lambda } x}{\sqrt{6}}\right) -10 \theta ^3 \lambda ^2 t^{3 \theta } \cosh \left( \frac{1}{2} \sqrt{\frac{3}{2}} \sqrt{\lambda } x\right) \cosh \left( \frac{\sqrt{\lambda } x}{\sqrt{6}}\right) \nonumber \\&\quad +5 \theta ^3 \lambda ^3 t^{3 \theta } \cosh \left( \frac{1}{2} \sqrt{\frac{3}{2}} \sqrt{\lambda } x\right) \cosh \left( \frac{\sqrt{\lambda } x}{\sqrt{6}}\right) -5 \theta ^4 \lambda ^3 t^{3 \theta } \cosh \left( \frac{1}{2} \sqrt{\frac{3}{2}} \sqrt{\lambda } x\right) \cosh \left( \frac{\sqrt{\lambda } x}{\sqrt{6}}\right) \nonumber \\&\quad 126 \theta ^3 \Gamma (\theta +1) t^{2 \theta } \cosh \left( \frac{1}{2} \sqrt{\frac{3}{2}} \sqrt{\lambda } x\right) -126 \theta ^2 \Gamma (\theta +1) t^{2 \theta } \cosh \left( \frac{1}{2} \sqrt{\frac{3}{2}} \sqrt{\lambda } x\right) \nonumber \\&\quad +66 \theta ^2 \lambda \Gamma (\theta +1) t^{2 \theta } \cosh \left( \frac{1}{2} \sqrt{\frac{3}{2}} \sqrt{\lambda } x\right) -6 \alpha ^3 \lambda \Gamma (\theta +1) t^{2 \theta } \cosh \left( \frac{1}{2} \sqrt{\frac{3}{2}} \sqrt{\lambda } x\right) \nonumber \\&\quad \frac{84 \theta ^3 \Gamma (\theta +1)^3 t^{2 \theta } \cosh \left( \frac{5 \sqrt{\lambda } x}{2 \sqrt{6}}\right) }{\Gamma (2\theta +1)}+\frac{84 \theta ^2 \Gamma (\theta +1)^3 t^{2 \theta } \cosh \left( \frac{5 \sqrt{\lambda } x}{2 \sqrt{6}}\right) }{\Gamma (2 \theta +1)} \nonumber \\&\quad \frac{20 \theta ^3 \lambda ^2 \Gamma (\theta +1) \Gamma (2 \theta +1) t^{3 \theta } \cosh \left( \frac{1}{2} \sqrt{\frac{3}{2}} \sqrt{\lambda } x\right) \cosh \left( \frac{\sqrt{\lambda } x}{\sqrt{6}}\right) }{\Gamma (3 \theta +1)}\nonumber \\&\quad \frac{6 \theta ^3 \Gamma (\theta +1) \Gamma (2 \theta +1) t^{3 \theta } \cosh \left( \frac{7 \sqrt{\lambda } x}{2 \sqrt{6}}\right) }{\Gamma (3 \theta +1)}+\frac{6 \theta ^3 \lambda \Gamma (\theta +1) \Gamma (2 \theta +1) t^{3 \theta } \cosh \left( \frac{7 \sqrt{\lambda } x}{2 \sqrt{6}}\right) }{\Gamma (3 \theta +1)}+\cdots \bigg ). \end{aligned}$$

Similarily,

70 $$\begin{aligned} \begin{aligned} \textsf{w}_0&=\textsf{w}(x,0),\\ \textsf{w}_1&=-\frac{5 (\lambda -1) \lambda \left( -\theta +\frac{\theta t^{\theta }}{\Gamma (\theta +1)}+1\right) \left( \tanh \left( \frac{\sqrt{\lambda } x}{2 \sqrt{6}}\right) -1\right) \text {sech}^2\left( \frac{\sqrt{\lambda } x}{2 \sqrt{6}}\right) }{24 \delta }\\ \textsf{w}_2&=\frac{5 (\lambda -1) \lambda ^2 \text {sech}^{10}\left( \frac{\sqrt{\lambda } x}{2 \sqrt{6}}\right) \left( \cosh \left( \frac{\sqrt{\lambda } x}{2 \sqrt{6}}\right) -\sinh \left( \frac{\sqrt{\lambda } x}{2 \sqrt{6}}\right) \right) }{18432 \delta ^2 \Gamma (\theta +1)^2 \Gamma (2 \theta +1) \Gamma (3 \theta +1)}\bigg ( 10 \theta ^3 (\lambda -1) \lambda ^2 \Gamma (2 \theta +1)^2 t^{3 \theta }\\&\quad \left( \cosh \left( \frac{5 \sqrt{\lambda } x}{2 \sqrt{6}}\right) -\sinh \left( \frac{5 \sqrt{\lambda } x}{2 \sqrt{6}}\right) \right) \theta ^2 \Gamma (\theta +1)^2 \Gamma (3 \theta +1) +t^{2 \theta }(355 \delta \cosh \left( \frac{\sqrt{\lambda } x}{2 \sqrt{6}}\right) \\&\quad +237 \delta \cosh \left( \frac{1}{2} \sqrt{\frac{3}{2}} \sqrt{\lambda } x\right) 20 \theta \lambda ^2 \cosh \left( \frac{5 \sqrt{\lambda } x}{2 \sqrt{6}}\right) \\&\quad +95 \delta \cosh \left( \frac{5 \sqrt{\lambda } x}{2 \sqrt{6}}\right) +20 \lambda ^3 \cosh \left( \frac{5 \sqrt{\lambda } x}{2 \sqrt{6}}\right) \\&\quad -20 \lambda ^2 \cosh \left( \frac{5 \sqrt{\lambda } x}{2 \sqrt{6}}\right) )+... \bigg ).\\&:\end{aligned} \end{aligned}$$

The final approximate two iterations series solution using $\textsf{ABC}$ fractional derivative for DLM for Case II will be:

71 $$\begin{aligned} \begin{aligned}{}&^\textsf{ABC}_2\textsf{p}(x,t)=\sum _{\mathfrak {j}=0}^{2} \textsf{p}_\mathfrak {j},\\&^\textsf{ABC}_2\textsf{w}(x,t)=\sum _{\mathfrak {j}=0}^{2} \textsf{w}_\mathfrak {j}. \end{aligned} \end{aligned}$$

Table 4 represents the precise, approximate and the absolute error results from solving the TFBZS (case II) using $\textsf{ABC}$ fractional derivative at $\theta =1$, $t=0.01$, $\delta =3$ and $\lambda =2$ for various *x* values.

**Table 4:** Precise and estimated solutions for the TFBZS (case II) at $\theta =1$, $t=0.01$, $\delta =2$ and $\lambda =2$.

*x*	$\textsf{p}(x,t)$			$\textsf{w}(x,t)$		
	Exact	Approx.	Abs. error	Exact	Approx.	Abs. error
− 10	0.993913	0.993914	8.43019 E−7	0.496957	0.496958	1.01806 E−6
− 6	0.941163	0.941170	7.17341 E−6	0.470582	0.470590	8.93003 E−6
− 2	0.582767	0.582778	1.09066 E−5	0.291384	0.291410	2.67739 E−5
2	0.0588943	0.0588787	1.56091 E−5	0.0294472	0.029448	7.98941 E−7
6	0.0009514	0.0009509	4.78581 E−7	0.0004757	0.0004757	2.19483 E−9
10	9.929 E−6	9.924 E−6	5.35382 E−9	4.964 E−6	4.964 E−6	2.94228 E−11

Considering the results we obtained from solving the two cases of initial conditions with different definitions, we notice that the Caputo results are very close to that results for Atangana-Baleanu-Caputo when the fractional order derivative $\theta =1$. Therefore, Table 5 illustrates a comparison for the results we obtained for the two cases when $\theta <1$.

**Table 5:** A comparison between the results obtained in the two cases at $\theta =0.8$, $t=0.01$, $\delta =2$ and $\lambda =3$.

*Cases*	*x*	Caputo		Atangana-Baleanu-Caputo	
		$\textsf{p}$	$\textsf{w}$	$\textsf{p}$	$\textsf{w}$
Case I	− 10	0.998413	0.001586	0.998725	0.001377
	− 6	0.973652	0.026347	0.979061	0.022411
	− 2	0.663884	0.336116	0.755336	0.240004
	2	0.042636	0.957363	0.098920	0.906818
	6	0.000229	0.999771	0.000656	0.999435
	10	8.250 E−7	0.999999	2.395 E-6	0.999998
Case II	− 10	0.998418	0.998424	0.999242	0.999968
	− 6	0.973725	0.973830	0.987167	0.999130
	− 2	0.664172	0.664982	0.785978	0.891803
	2	0.042397	0.042652	0.072723	0.100651
	6	0.000227	0.000229	0.000419	0.000656
	10	8.171 E−7	8.250 E−7	1.517 E−6	2.395 E−6

## Graphic representations

Graphic representations offer a visual context that enhances the comprehension of data and results. They provide an immediate and intuitive understanding of the relationships, and patterns present in the data, making it easier for researchers and readers to grasp the significance of the findings.

**Figure 1:**
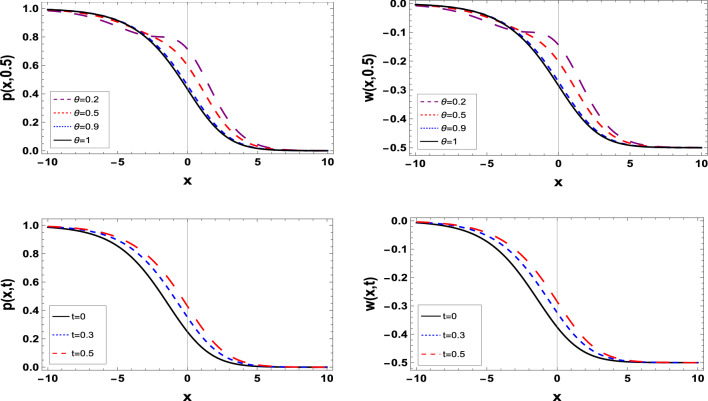
The estimated solution of the TFBZS (Case I) under $\textsf{C}$ fractional derivative presented in Eq. (51) at $\delta =1$ and $\lambda =1.5$.

**Figure 2:**
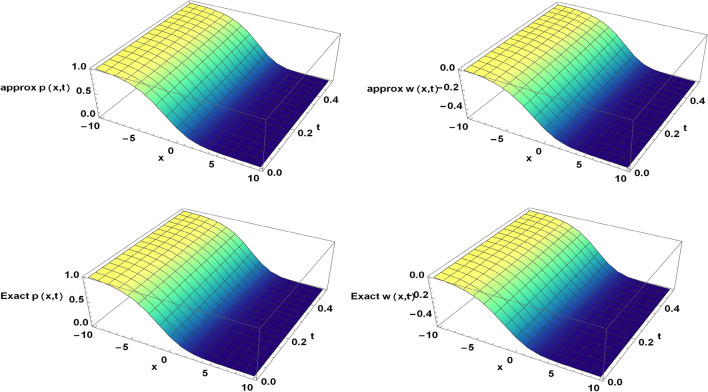
The approximate and exact solutions of the TFBZS (Case I) under $\textsf{C}$ fractional derivative presented in Eq. (51) at $\delta =1$ and $\lambda =1.5$.

**Figure 3:**
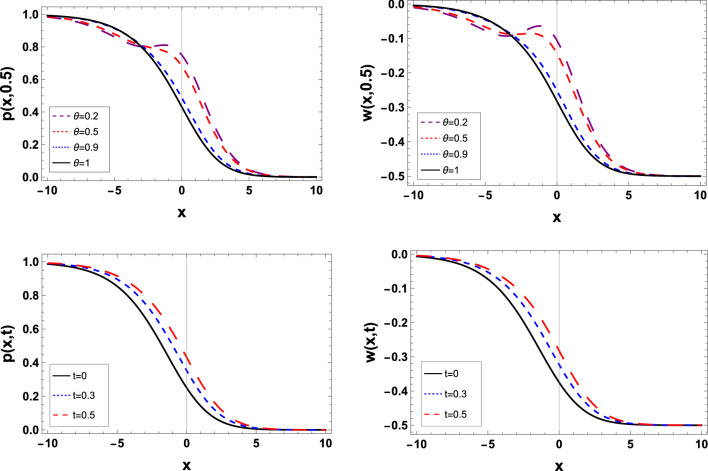
The estimated solution of the TFBZS (Case I) under $\textsf{ABC}$ fractional derivative presented in Eq. (62) at $\delta =1$ and $\lambda =1.5$.

**Figure 4:**
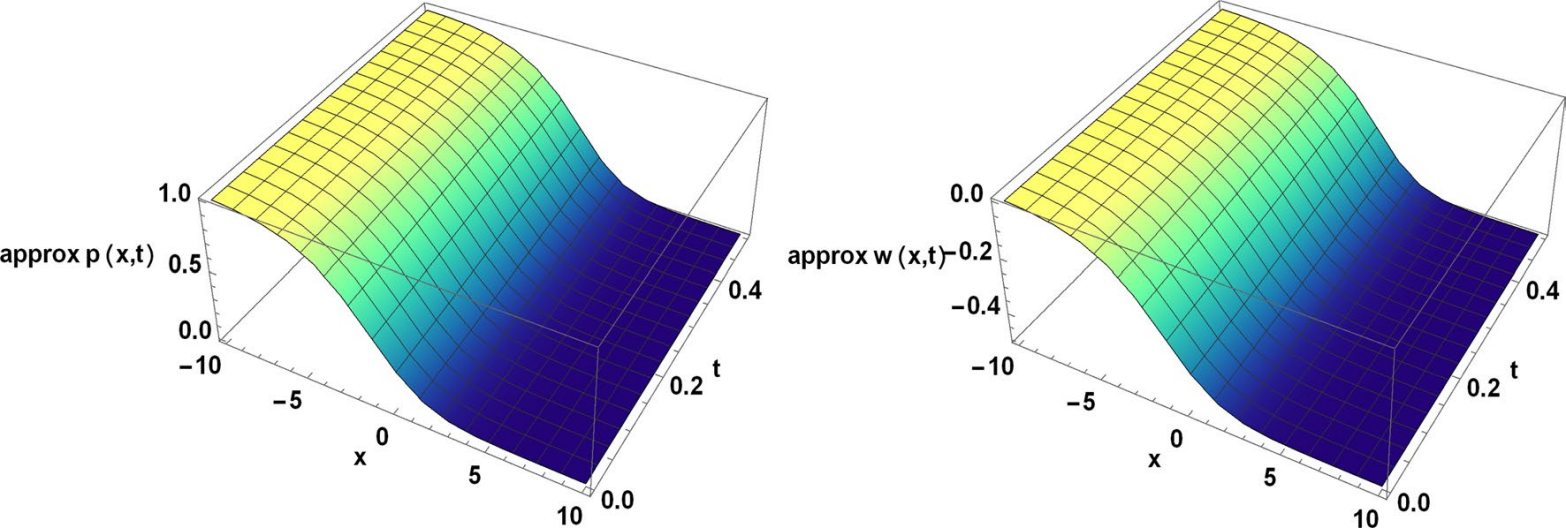
The approximate solution of the TFBZS (Case I) under $\textsf{ABC}$ fractional derivative presented in Eq. (62) at $\delta =1$ and $\lambda =1.5$.

**Figure 5:**
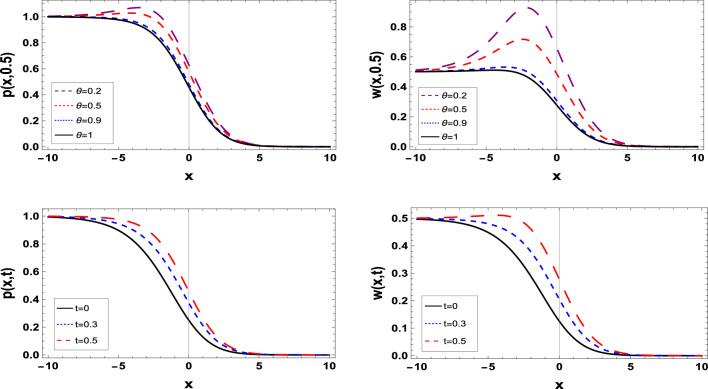
The estimated solution of the TFBZS (Case II) under $\textsf{C}$ fractional derivative presented in Eq. (67) at $\delta =2$ and $\lambda =2$.

**Figure 6:**
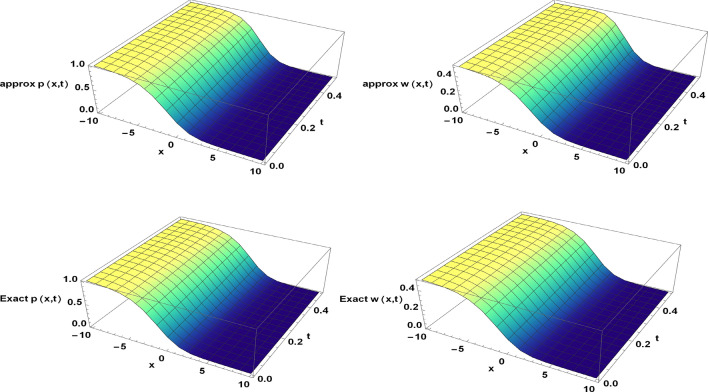
The exact and approximate solution of the TFBZS (Case II) under $\textsf{C}$ fractional derivative presented in Eq. (67) at $\delta =2$ and $\lambda =2$.

**Figure 7:**
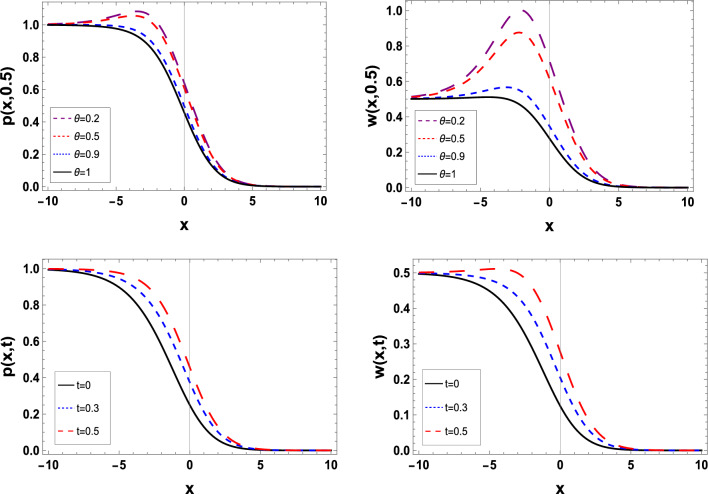
The estimated solution of the TFBZS (Case II) under $\textsf{ABC}$ fractional derivative presented in Eq. (71) at $\delta =2$ and $\lambda =2$.

**Figure 8:**
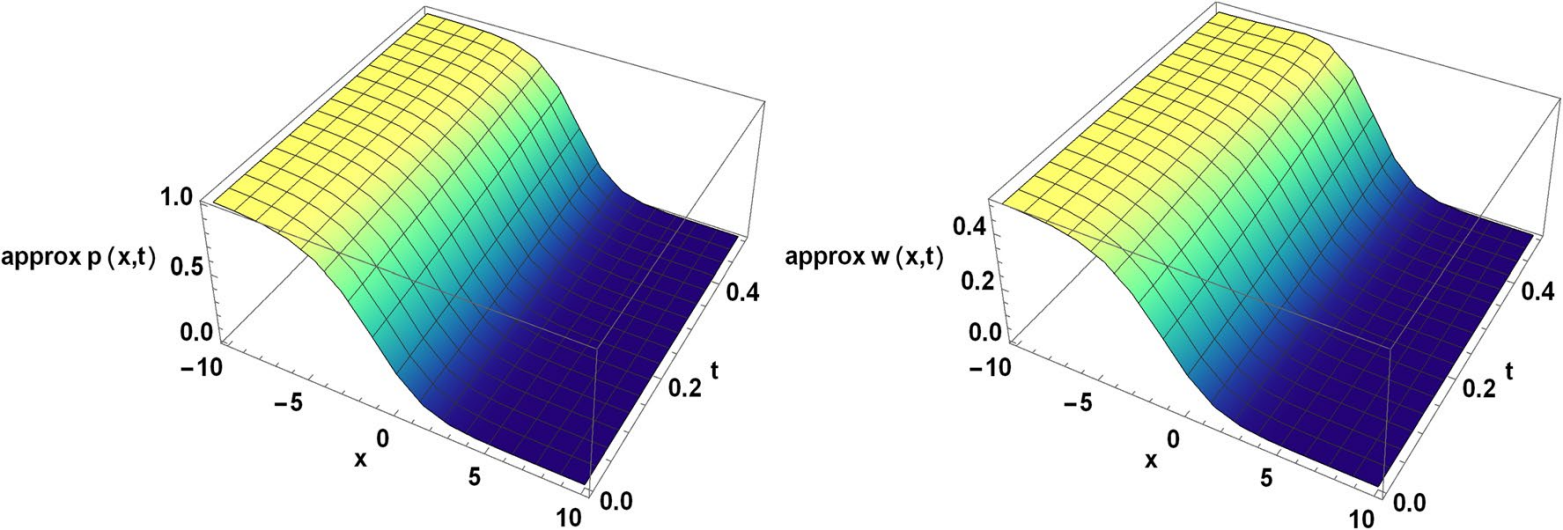
The approximate 3D solution of the TFBZS (Case II) under $\textsf{ABC}$ fractional derivative presented in Eq. (71) at $\delta =2$ and $\lambda =2$.

Two and three dimensions graphs for the obtained solutions are presented to visualize the behavior of the TFBZS using two different cases of initial conditions each case was dealt with in two definitions of fractional calculus $\textsf{C}$ and $\textsf{ABC}$. Figure 1 represents the two-dimensional visualization at different fractional order parameter values $\theta$ with fixed time $t=0.5$, and at $\theta =1$ for several steps of time, this in for the first case of initial conditions using DL Caputo. Figure 2 shows the three-dimensional approximate and exact representation for case I using $\textsf{C}$, the graphs shows great coincides between exact and estimated solution which reflect the efficiency of the used method. Figure 3 clarify the 2D solution of the TFBZS (case I) using $\textsf{ABC}$ DLM for the two unknown functions $\textsf{p}$ and $\textsf{w}$ at different values of $\theta$ with fixed time and at several stages of time with fixed $\theta =1$. Figure 4 shows the approximate solution in three-dimensions of the TFBZS at $\delta =1$ and $\lambda =1.5$. The graphs using $\textsf{C}$ and $\textsf{ABC}$ also very close to each other, this means that either using $\textsf{C}$ DLM or $\textsf{ABC}$ DLM, we obtain high solution accuracy. Figure 5 shows the estimated solution of using DLM using $\textsf{C}$ for case II of initial conditions. Figure 6 represents the exact and approximate solution in three-dimensions for case II, its clear that, the estimated solution is nearly the exact solution at the same values of parameters. Figures 7 and 8 represent the obtained solution in two- and three-dimensions using $\textsf{ABC}$ DLM for case II of initial conditions. All the represented graphs show coincides between the estimated and exact solution which ensures the validity of the DLM for solution.

## Conclusion

In this study, we obtain an approximate series solution for the TFBZS using DLM under varying initial conditions. Each initial condition was examined using both Caputo and Atangana-Baleanu Caputo fractional derivatives. The results obtained showcased an impressive level of accuracy, with errors consistently maintained at a remarkably low magnitude. Furthermore, we conducted a thorough investigation into the existence and uniqueness aspects of the solution, establishing a robust foundation for the validity of our approach. In order to understand the behavior of the solution, we present two and three dimensional graphs to show the impact of the time and the fractional derivative of the solution. The graphs of approximate and exact solution demonstrate a close resemblance, indicating the accuracy of the obtained solutions.

For the future directions, we envision expanding the application of the DLM to other complex fractional systems with higher-order fractional derivatives that analyze a real world applications. This can involve exploring the applicability of the method in multi-dimensional fractional systems.

## Use of AI tools declaration

The authors confirm that they did not utilize any Artificial Intelligence (AI) tools in the development of this article.

## Data Availability

The data that support the findings of this study are available from the corresponding author upon reasonable request.
